# *Acinetobacter radioresistens* Bacteremia in Complicated Pneumonia. A Case Report and Literature Review

**DOI:** 10.1002/ccr3.71481

**Published:** 2025-11-18

**Authors:** Sara Campana, Francesco Giannone, Marco Torri, Carlo Rostagno

**Affiliations:** ^1^ Medicina Interna 3 AOU Careggi Firenze Italy; ^2^ Medicina Interna 3, AOU Careggi Firenze Dipartimento Medicina Sperimentale e Clinica Università Degli Studi di Firenze Firenze Italy

**Keywords:** Acinetobater sp., antibiotic resistance, carbapenemase, pneumonia

## Abstract

*Acinetobacter radioresistens* , a ubiquitous non‐pathogen microorganism, is the source of the class D carbapenemase OXA‐23 being therefore a potential disseminator of resistance genes in Acinetobacter spp. At present few cases of human infections have been reported in the literature, all sensible to beta‐lactams. The present paper reports for the first time an infection by *Acinetobacter radioresistens* showing resistance to sulfamethoxazole‐trimethoprim and intermediate sensitivity to meropenem and ciprofloxacin.

## 1 Introduction

Acinetobacter spp. are aerobic, Gram‐negative coccobacilli [1] that grow at 20°C–30°C on common laboratory media [2, 3]. The genus *Acinetobacter* includes at least 38 genomic species, which are ubiquitous, and most of them are part of the normal skin microflora [4]. These microorganisms can cause community‐acquired and primary care‐associated infections because of their ability to acquire antimicrobial resistance genes, leading to multidrug resistance. *Acinetobacter baumannii* is the best‐known member of this family of pathogens; it primarily causes bacteremia, pulmonary, urinary tract, and surgical wound infections. Invasive procedures (use of mechanical ventilation, central venous or urinary catheters) and broad‐spectrum antimicrobials are the main factors that increase the risk of infection [5]. *Acinetobacter radioresistens* has been originally isolated from gamma‐sterilized cotton, which resists extreme environmental conditions such as desiccation, UV radiation or vapor, and hydrogen peroxide [6]. It is found on the human skin and considered to be part of the human flora; however, rarely is it responsible for bacteremia or pneumonia [6]. *Acinetobacter radioresistens* is a potential disseminator of resistance genes and it is the source of the class D carbapenemase OXA‐23; therefore, its immediate and accurate identification is critical since it could act as a silent reservoir for carbapenemase resistance in hospitalized patients [7]. *Acinetobacter radioresistens* has been reported to cause less than 10% of all Acinetobacter infections. Nevertheless it has been described as the most common species of its genus in hospital environmental samples. Biochemical‐based methods may lead to misidentification and some of the previously reported infections due to *Acinetobacter* species may have been effectively due to *A. radioresistens* [8]. Few cases of *Acinetobacter radioresistens* isolation from biological specimens are reported in the literature; the first occurred in 2001 in an HIV patient [9]. There was a slight male prevalence a wide age distribution (32–83 years). All patients had relevant comorbidities. Pulmonary involvement was present in almost all patients. The microorganism was more frequently isolated in blood cultures; in the other case in bronchoalveolar lavage [10, 11, 12, 13, 14, 15, 16]. Co‐infection with other microorganisms has been reported. Recently, two cases of endocarditis secondary to infection by *Acinetobacter radioresistens* and *Enterococcus casseliflavus were described*, the first, and *Microbacterium paraoxydans the second* [17, 18]. We report a case of a 72‐year‐old male with several comorbidities and *A. radioresistens* pneumonia complicated by influenza and fungal superinfection.

## 2 Case History/Examination

A 72‐year‐old man was admitted to the emergency department for a 10‐day history of cough, dyspnea, fever with chills, and diffuse arthralgia. His medical history included pulmonary emphysema in a former smoker (100 packs/year) and hypertension. The patient had no recent hospitalizations and was vaccinated for COVID‐19.

On presentation, the patient was markedly tachypneic (30 breaths/min), BP 124/75 mmHg, HR 80 bpm, SpO_2_ 90%, TC 37.2°C, and chest physical examination showed a diffuse decrease in breath sounds. Crackles were appreciable in the right basal and left mid‐basal grounds, Laboratory findings revealed neutrophilia and an increase in D‐dimer and C‐reactive protein (C‐reactive Protein 115 mg/L). Serum lactic acid concentration was 1.1 mmol/L, procalcitonin 0.29 ng/mL, and creatinine 1.3 mg/dL.

**FIGURE 1:**
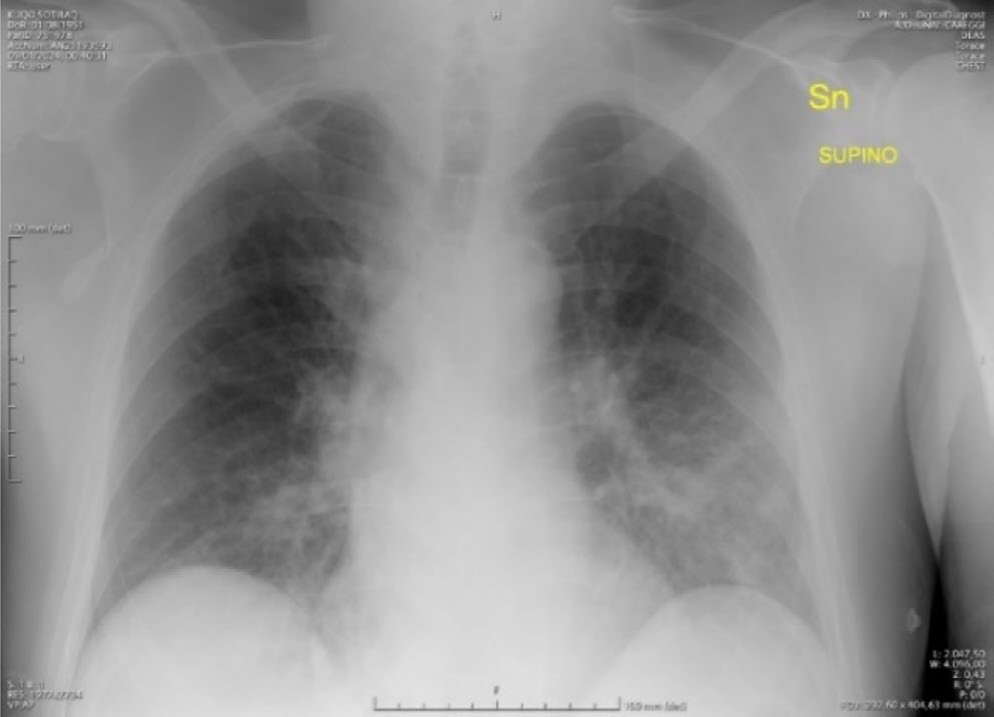
Chest x‐ray at ED admission showing infiltrates at the left diaphragmatic paracardiac site.

In the ER a chest X‐ray showed mild parenchymal infiltrates at the left diaphragmatic paracardiac site (Figure 1).

## 3 Differential Diagnosis, Investigations and Treatment

Empiric treatment with intravenous ceftriaxone (2 g daily), azithromycin (500 mg daily), triple inhalation therapy, and i.v. corticosteroids was administered at arrival; the condition quickly deteriorated and required noninvasive ventilation (CPAP 8 cmH_2_O, FiO_2_ 60%). The critical condition did not allow for the performance of bronchoscopy due to the high procedural risk. Antibiotic therapy was changed to piperacillin‐tazobactam.

The research for Mycoplasma, Quantiferon, HIV Ab, Parvovirus, CMV, EBV, B‐D‐glucan, other than nasopharyngeal swab for Sars Cov 2 and urinary antigens for Legionella and Pneumonia was negative. As well two swabs for influenza A and B were found negative. Immunophenotype on peripheral blood showed normal B/T balance and CD4/CD8 ratio and mild depression of NK cells ANCA‐ANA‐ENA‐ anti‐dsDNA autoantibody was negative.

**FIGURE 2:**
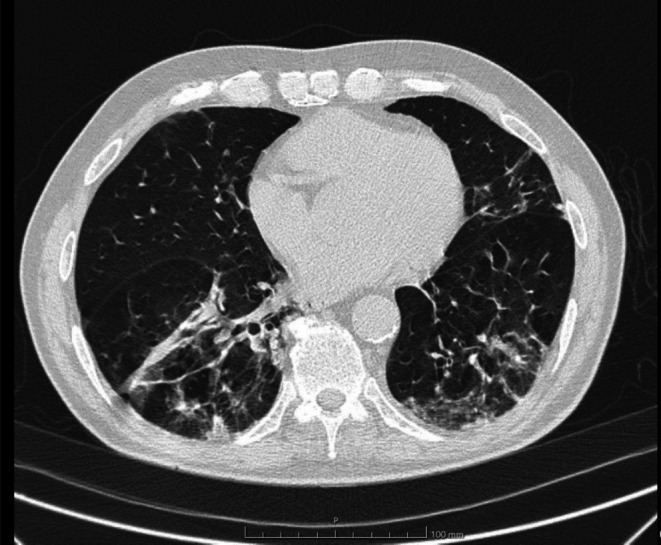
CT scan—extensive areas of consolidation, with peribronchial distribution and subpleural OOP‐like aspects, which are associated with post‐obstructive atelectatic aspects in the apical segment of the LIL, fuzzy “tree‐in‐bud” centrilobular micronodularities, and bronchial wall thickening.

Due to the persistence of fever despite antibiotic therapy and severe respiratory failure, a chest CT scan was performed (Figure 2); the CT showed extensive areas of consolidation, with peribronchial distribution and subpleural OOP‐like aspects, which are associated with post‐obstructive atelectatic aspects in the apical segment of the LIL, fuzzy “tree‐in‐bud” centrilobular micronodularities, and bronchial wall thickening.

Blood cultures were positive for *Acinetobacter radioresistens* . Antibiogram showed resistance to sulfamethoxazole–trimethoprim and intermediate sensitivity to meropenem and ciprofloxacin (Table 1). Antibiotic treatment with meropenem 2 g × 3/day and amikacin 1 g/day was started. Treatment was conducted for a total of 13 days, with progressive resolution of the fever.

**TABLE 1:** Antibiogram of *A. radioresistens* isolated in blood cultures.

	Antibiotic	S	MIC
*Acinetobacter radioresistens*	Amikacine	S	≤ 4
	Ciprofloxacin	I	1
	Gentamicine	S	2
	Meropenem	I	8
	Sulphamethoxazole‐Trimethoprim	R	8
	Colistin	S	≤ 0.5
	Tigecyclin	S	< 0.25

## 4 Conclusion and Results

**FIGURE 3:**
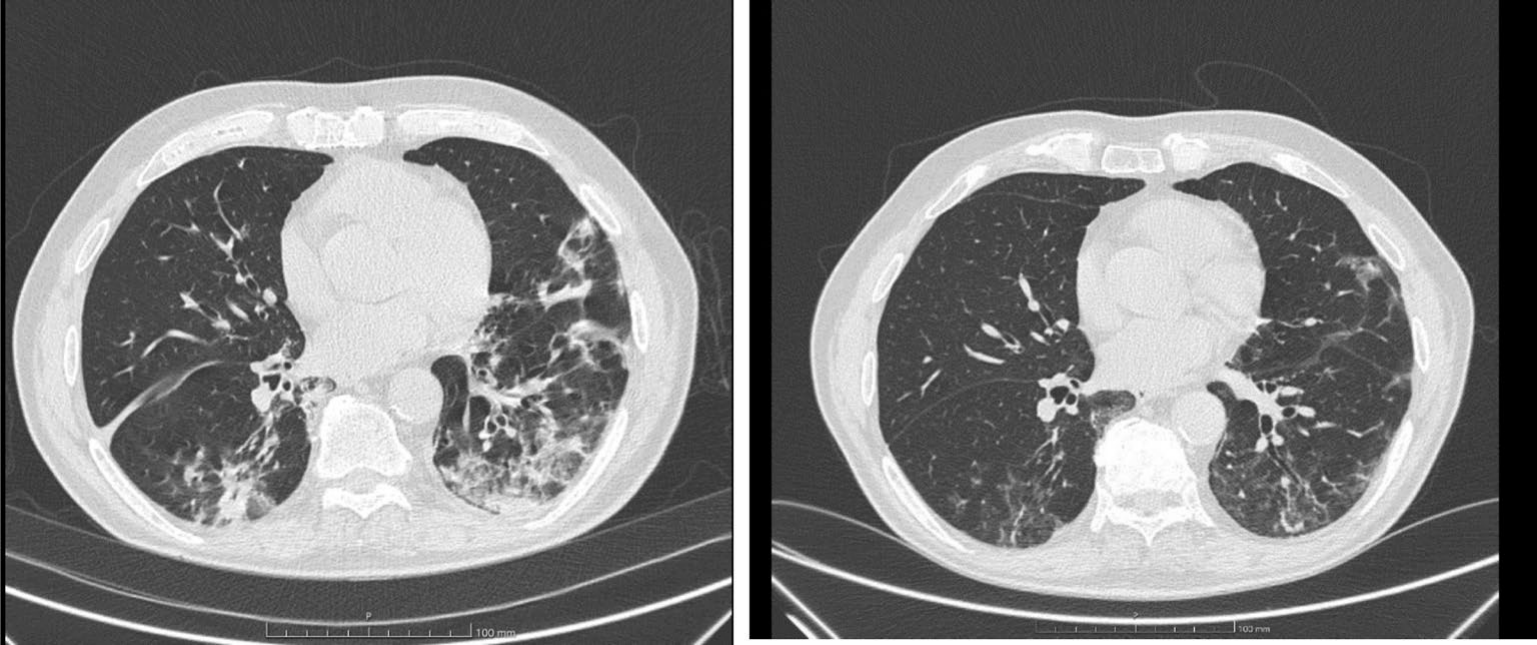
(a) Chest CT scan after 10 days of therapy; BAL positivity for fungal overinfection and A influenza did not show significant improvement (b) chest TC scan at 2 months follow‐up showing almost complete resolution of radiological abnormalities.

Noninvasive ventilation could be weaned; however antibiotic treatment did not result in a definite improvement of respiratory failure with persistent desaturation at minimal efforts. A second CT scan (Figure 3a) did not show resolution of lung consolidation. Fibro bronchoscopy was scheduled, and BAL was positive for aspergillus and influenza virus type A. Antiviral therapy with oseltamivir 75 mg × 2 for 7 days and antifungal isavuconazole (loading dose 200 mg t.i.d then 200 mg/day for 1 month) was started.

Antivirals were discontinued after 14 days, maintaining long‐acting antifungal therapy The patient underwent respiratory rehabilitation with recovery of functional autonomy about 1 month after hospitalization.

At follow‐up control, 2 months after admission, the patient was in good clinical condition and antifungal therapy was withdrawn after control CT scan (Figure 3b).

## 5 Discussion

The most frequently described Acinetobacter is *A. baumannii*, whose sensitivity profile is variable. In recent years, it has gained high relevance as a pathogen in nosocomial infections (particularly in intensive care units), also for its ability to develop multidrug resistance that has led to carbapenem‐resistant healthcare‐associated infection outbreaks.

*Acinetobacter radioresistens* although being a ubiquitous non‐pathogen microorganism, rarely causes severe sepsis, pneumonia and respiratory failure. The main concern with the microorganism is that it may be a source of the OXA‐23 gene, a class D carbapenemase, which can confer carbapenem resistance in *A. baumannii* .

In literature infections by *A. radioresistens* are more frequently acquired in the community and associated with comorbid conditions (mainly COPD, heart failure+ and malignancies). These were reported to occur in a wide range of ages (32–85 years) with a mild male prevalence (Table 2). The isolation source was blood in almost all patients and tracheobronchial secretions in four. A high antimicrobial sensitivity has been previously reported in the literature, and all patients were treated with beta‐lactams. Fifty percent were discharged alive from the hospital, and two patients or 16% died. The outcome in the other patients was unknown.

**TABLE 2:** Reported cases of A. radioresistens infection.

Case	Age and sex	Comorbid conditions	Source	Co‐infection	Outcome
Visca et al. [9]	32 F	Ye	Blood	Yes	Survived
Savov et al. [10]	85 M	Yes	Tracheobronchial	?	Died
Brady et al. [11]	53 F	Yes	Blood	No	Survived
	60 M	Yes	Blood	No	Survived
Verma et al. [12]	61 M	Yes	Blood	No	Unknown
Wang et al. [13]	71 F	Yes	Blood; tracheobronchial	No	Died
Lopes et al. [14]	73 M	Yes	Blood; bronchial	No	Unknown
Tan et al. [15]	55 M	N/A	Blood; bronchial	No	Unknown
Lazarev et al. [16]	83 M	Yes	Blood	No	Survived
Motie et al. [17]	63 M	Yes	Blood	Yes	Survived
Pinchman et al. [18]	60 F	Yes	Blood	Yes	Unknown
Present case	72 M	Yes	Blood	Yes	Survived

In our patient the antibiogram reported resistance to beta‐lactams and trimethoprim‐sulfamethoxazole and intermediate sensitivity to meropenem and ciprofloxacin. It was therefore necessary to shift therapy to amikacin associated with high‐dosage meropenem. Antibiotic combination therapy rather than monotherapy allows for an increase in the spectrum of antibiotic activity and assures a possible synergistic effect [17]. Combination therapy moreover may avoid—or at least reduce—the onset of bacterial resistance. We chose combination therapy including meropenem despite intermediate sensitivity in the antibiogram to take advantage of the synergic action of different mechanisms. Carbapenem‐sparing agents such as cefiderocol or i.v. fosfomycin may be valuable alternatives [18, 19] in the treatment of multidrug‐resistant infections but were not initially considered in our patient. Cefiderocol a siderophore cephalosporin, enters bacterial walls through iron–siderophore complex channels in an energy‐dependent manner [19]. Antimicrobial effects are related to the consequent inhibition of cell wall synthesis.

Co‐infection or superinfection by influenza A and Aspergillus further complicated the clinical course of the disease. We cannot state whether these represent a co‐infection or a complication of *A. radioresistens* infection since fibro bronchoscopy was not possible due to the clinical conditions at admission and both influenza A and Aspergillus were found in BAL. As recently reported co‐infection was demonstrated in two cases of *Acinetobacter radioresistens* endocarditis [20, 21]. Immunodepression, as in the case reported by Visca et al. [9] in a patient with HIV, may have favored fungal superinfection.

The difficulties in microbiological identification [21, 22, 23, 24] may lead to an underestimation of the prevalence of *A. radioresistens* in human infections. The main concern with A. radioresistens outbreaks is related to its ability to be a potential reservoir for carbapenem resistance. Class D resistance to carbapenems (OXA enzyme type) is usually harbored by plasmids. *A. radioresistens* may be the source of the *bla*_*OXA‐23*_ gene which confers carbapenem resistance to *A. baumannii*. *A. radioresistens* therefore may be a silent reservoir for carbapenem resistance.

In our report *A. radioresistens* showed resistance to sulfamethoxazole–trimethoprim and intermediate sensitivity to meropenem and ciprofloxacin suggesting that changes in sensitivity characteristics may lead to more difficult management of infections related to this microorganism. Combination therapy should be started as soon as possible to take advantage of different synergic effects.

## Author Contributions

**Sara Campana:** conceptualization, resources. **Francesco Giannone:** resources, writing – original draft. **Marco Torri:** writing – review and editing. **Carlo Rostagno:** writing – review and editing.

## Consent

Written informed consent was obtained from the patient to publish this report in accordance with the journal's consent policy.

## Conflicts of Interest

The authors declare no conflicts of interest.

## Data Availability

The clinical course is recorded in electronic medical records of our hospital.
